# Adipose‐derived stem cell spheroids are superior to single‐cell suspensions to improve fat autograft long‐term survival

**DOI:** 10.1111/jcmm.17082

**Published:** 2022-02-11

**Authors:** Sanae El Harane, Stéphane Durual, Thomas Braschler, Dominik André‐Lévigne, Nicolo Brembilla, Karl‐Heinz Krause, Ali Modarressi, Olivier Preynat‐Seauve

**Affiliations:** ^1^ Department of Pathology and Immunology Faculty of Medicine University of Geneva Geneva Switzerland; ^2^ Laboratory of Biomaterials Faculty of Dental Medicine University of Geneva Geneva Switzerland; ^3^ Division of Plastic, Reconstructive and Aesthetic Surgery Geneva University Hospitals University of Geneva Geneva Switzerland; ^4^ Laboratory of Therapy and Stem Cells Geneva University Hospitals Geneva Switzerland; ^5^ Department of Medicine Faculty of Medicine University of Geneva Geneva Switzerland

**Keywords:** adipose‐derived stem cells, fat transplantation, regenerative medicine, spheroids

## Abstract

Autologous fat transplantation is a widely used procedure for surgical reconstruction of tissues. The resorption rate of this transplantation remains high and unpredictable, reinforcing the need of adjuvant treatments that increase the long‐term stability of grafts. Adipose‐derived stem cells (ASC) introduced as single cells in fat has been shown clinically to reduce the resorption of fat grafts. On the other hand, the formulation of ASC into cell spheroids results in the enhancement of their regenerative potential. In this study, we developed a novel method to produce highly homogeneous ASC spheroids and characterized their features and efficacy on fat transplantation. Spheroids conserved ASC markers and multipotency. A regenerative gene expression profile was maintained, and genes linked to autophagy were upregulated whereas proliferation was decreased. Their secreted proteome was enriched in comparison with single‐cell ASC suspension. Addition of spheroids to fat graft in an animal model of transplantation resulted in a better graft long‐term stability when compared to single ASC suspension. In conclusion, we provide a novel method to manufacture homogenous ASC spheroids. These ASC spheroids are superior to ASC in single‐cell suspension to improve the stability of fat transplants, reinforcing their potential in reconstructive surgery.

## 1 INTRODUCTION

Autologous fat grafting is widely used worldwide for tissue augmentation in a variety of reconstructive applications: breast reconstruction after cancer treatment, scar correction after burn or trauma, correction of congenital malformations. Lipoaspirates from patients own fat tissue are the ideal source of fat grafts because of fat biocompatibility, natural‐looking, absence of immunogenicity and easy availability. The major obstacle of fat grafting is an unpredictable and often low graft survival, with a lack of vascularization and resorption rates ranging from 25% to 80%.^1^, ^2^, ^3^, ^4^

Adipose‐derived stem cells (ASC) reside in the stromal fraction of adipose tissue.^5^ They harbour self‐renewing capacities allowing their ex vivo expansion, can differentiate in vitro into many cell types and, importantly, produce a large spectrum of factors involved in tissue regeneration and healing. The International Society for Cellular Therapy and International Fat Applied Technology Society established the criteria, which determine ASC in vitro ^5^: (i) adherence to plastic surface in standard culture conditions (ii) defined expression of surface antigens while hematopoietic markers must be absent (iii) ability to multilineage differentiation into adipocytes, osteocytes and chondrocytes in vitro. The ability of ASC to produce factors involved in tissue regeneration makes them highly attractive for cell therapy applications. Although their differentiation capacities observed in vitro are now considered to be absent in vivo after injection,^6^, ^7^ therapeutic effects are attributed to their secretome favouring local healing. The ASC secretome has been extensively studied^8^ and contains factors enhancing the regenerative process.

Recent studies have shown that fat grafts enriched with ex vivo expanded ASC in single‐cell suspension markedly improved residual graft volume and histological appearance^9^, ^10^ due to their regenerative secretome. Plastic surgeons introduced clinically the technique of fat graft enrichment with ASC in single‐cell suspension. Compared with the control grafts, the ASC‐enriched fat grafts (injected in the posterior part of upper arms) had significantly higher residual volumes.^11^ The procedure had excellent safety, reinforcing the prospect of ASC use in clinical settings.

On the other hand, cell spheroids have gained considerable popularity.^12^, ^13^, ^14^ These structures allow a 3D cell‐to‐cell and cell‐to‐matrix interactions which guarantee cell functions, nutrient and oxygen gradients closer to the in vivo situation, as compared to 2D cell monolayers growing on plastic or single‐cell suspensions. Numerous reports demonstrate that the aggregation of mesenchymal stem cells or ASC into spheroids results in the enhancement of their therapeutic potential.^13^, ^15^, ^16^ Compared to their single‐cell suspensions equivalents, MSC or ASC spheroids showed increased regenerative properties with a higher production of angiogenic and immunomodulatory factors.^17^, ^18^, ^19^, ^20^, ^21^ In vivo, by using models of kidney degeneration,^22^ hepatic fibrosis^23^ or failure,^24^ urinary tract degeneration,^25^ wounds^18^, ^26^, ^27^ and lungs disease,^28^ MSC or ASC spheroids showed a higher regenerative potential than single‐cell suspensions. They notably displayed better survival in ischaemic conditions^29^ and higher resistance to oxidative stress‐induced apoptosis.^30^

In this study, we developed a novel method to manufacture stable and highly homogeneous human ASC spheroids suitable for cell therapy and compared the regenerative effects of single ASC suspensions versus spheroids on fat transplantation.

## 2 MATERIAL AND METHODS

### 2.1 ASC culture and differentiation

Different human ASC lines were prepared from the fat of patients as described (Uckay, I., et al., J Stem Cell Res Ther, 2019. 9). Cells were cultured in Dulbecco's Modified Eagle Medium DMEM (4.5 g/L glucose, L‐Glutamine) supplemented with 10% of human platelet lysate (Stemulate, Cook Regentek), 1% penicillin and streptomycin (Thermo Fisher) at 37°C and under 5% CO2. This study was conducted according to the approval by local ethical committee of the University Hospitals of Geneva, Switzerland (2020–01102 and NAC 14–183). Written informed consent was obtained from each individual. The multipotent differentiation into adipocytes, osteocytes and chondrocytes was performed on a monolayer of ASC directly derived from ASC spheroids by using the Human Mesenchymal Stem Cell Functional Identification Kit (R&D Systems) according to the supplier's instructions.

### 2.2 Moulding and 3D printing of pads

Pads were created by moulding Aggrewell‐800 plates (Stemcell) with a Sylgard 184 (Sigma‐Aldrich) silicone elastomer kit according to the supplier's instructions (Dow). In other experiments, the pads were 3D‐printed. They were designed by using an open‐source parametric 3D modeler (FreeCAD 0.18). Generated designs were then exported as STL format files and transduced into a G‐code file with the software Autodesk Netfab2020. The resulting file was imported into a digital light processing (DLP) 3D printer (Rapidshape P30 series, Straumann^®^) with a UV385 nm high power led and a HD resolution of 1920 × 1080 px. An acrylic resin (Sheraprint model plus sand UV) was used to print the models with a layer thickness of 50 µm. Once printed, the models were washed two times for 6 min in isopropanol in an ultrasonic bath (28–34 kHz) at room temperature. Once dried, the models were further cured in a curing unit (Shera flashlight‐+3D): 2 cycles of 2000 flashes (100 W/280–700 nm/10 flashes per seconds) before sterilization and moulding.

### 2.3 Spheroids formation

For the moulding of microwells in agarose, heated ultrapure agarose (LE, Analytical Grade, Promega) at 2.5% in ultrapure water was deposited on Milicell inserts (Merckmillipore, 0,4 µm). The pad was then immediately placed in the heated agarose. After 10 min for complete polymerization of agarose, the pad was gently removed. To form the spheroids, the ASC suspension in their culture medium was filtered through a cell strainer (Corning, 40 µm) and 1 ml of suspension was deposited on agarose/inserts in 6‐well plates. The plate was then centrifuged at 100 *g* for 5 min to force aggregation of ASC in microwells. To establish air/liquid interface conditions, the residual medium in the insert was removed by aspiration with a 0.5 × 16 mm needle and 1 ml of fresh medium were added in each well of the 6‐well plate.

### 2.4 Determination of spheroids viability

The viability of the spheroids was controlled with a fluorescent labelling kit (Ibidi) with fluorescein diacetate (FDA) and propidium iodide (PI), according to the supplier's instructions.

### 2.5 Immunofluorescence, histological colorations

Spheroids were washed in PBS and then fixed with a 4% paraformaldehyde solution for 20 min at room temperature. The spheroids were then rinsed in PBS and replaced in PBS supplemented with bovine serum albumin 1%, Triton X‐100 0.3%. The spheroids were then incubated overnight at +4°C with the primary anti‐Stro1 Mouse IgM (Life Technologies). After three washes in PBS, incubation with Alexa555 secondary anti‐Stro1 Mouse IgM (Invitrogen) was performed for 90 min. Again, a step of three washes was performed before the nucleus staining with DAPI (300 nM). After three successive washes in PBS, cells were rinsed in pure water and fixed in FluorSave mounting medium (Calbiochem). Histological coloration with hemalun‐eosine was done according to the standard protocol. Antibodies used were a polyclonal rabbit anti‐perilipin‐1 (abcam) and a rabbit polyclonal anti‐mouse CD31 (Abcam).

### 2.6 Molecular biology

Microarray analysis was performed with an Affymetrix chip targeting 21,448 mRNAs on the extracted RNA (Complete GeneChip^®^ Instrument System, Affymetrix) in biological triplicates. These biological triplicates were established from RNAs extracted from 3 independent ASC lines, each line being derived from one patient. Isolation of total RNA was performed by using RNeasy kit from Qiagen according to the manufacturer's instructions. RNA concentration was determined by a spectrometer (Thermo Scientific™ NanoDrop 2000), and RNA quality was verified by 2100 bioanalyzer (Agilent). The biological triplicates allowed the calculation, between the two conditions (2D versus spheroids), of a fold change. This fold change analysis was at the same time associated with a statistical analysis calculating a corrected *p* value (*p* < 0.05, non‐parametric Mann‐Whitney t test). Corrected *p* values (= False Discovery Rates, FDR) that were not statistically significant were systematically removed from the analysis. The heatmap and principal component analysis were performed by using the TAC4.0.1.36 software (Biosystems) associated with the Complete GeneChip^®^ Instrument System (Affymetrix) and the heatmap package (https://cran.r‐project.org/web/packages/pheatmap/index.html) with default settings. A functional analysis of the gene families most regulated by ASC in spheroids was performed using an algorithm combining the statistical software R and the Gosummaries package. All regulated genes with a fold change > 2 or <−2 and with statistical significance (FDR < 0.05) were taken into account (Table S1). Firefly luciferase was introduced in lentivectors under the control of the ubiquitous promoter of short elongation factor (EFS), and cells were transduced, as described previously.^31^

### 2.7 Mass spectrometry

Two million cells in 2D or spheroids were washed with a serum‐free DMEM (Thermo Fisher) and cultured overnight at 37°C in 1 ml of medium. Medium was collected and centrifuged at 500 *g* for 10 min to remove residual cells and free nuclei. Proteins were precipitated, digested and peptides were analysed by nanoLC‐MSMS using an easynLC1000 (Thermo Fisher) coupled with a Q Exactive HF mass spectrometer (Thermo Fisher). Database searches were performed with Mascot (Matrix Science) using the Human Reference Proteome database (Uniprot). Data were analysed and validated with Scaffold (Proteome Software) with 1% of protein FDR and at least 2 unique peptide per protein with a 0.1% of peptide FDR. Peaklist (MGF file format) were generated from raw data using the MS Convert conversion tool from ProteoWizard. The peaklist files were searched against the Human Reference Proteome database (Uniprot, release 2019–09, 20660 entries) combined with an in‐house database of common contaminant using Mascot (Matrix Science, London, UK; version 2.5.1). The Mascot searches were validated using Scaffold 4.10.0 (Proteome Software). Peptide identifications were accepted if they could be established at greater than 5.0% probability to achieve an FDR less than 0.1% by the Peptide Prophet algorithm^32^ with Scaffold delta‐mass correction. Protein identifications were accepted if they could be established at >22.0% probability to achieve an FDR < 1.0% and contained at least 2 identified peptides. Protein probabilities were assigned by the Protein Prophet algorithm.^33^ Proteins were annotated with GO terms from NCBI.^34^ Experiments were performed in biological duplicates, established from 2 independent ASC lines from two different patients. Functional protein class analysis was done by PANTHER (Protein ANalysis THrough Evolutionary Relationships, www.pantherdb.org) classification system and a stringDB analysis (using STRING database, www.string‐db.org) of known and predicted protein‐protein interactions.

### 2.8 Flow cytometry

Cells were incubated in 100 µl of PBS supplemented with 10% of bovine serum albumin with each dilution of the antibodies, all from Abcam, according to the concentration recommended by the supplier (μl/cells). Isotype controls (mouse IgG1) with the same fluorochrome were used. The cells and antibodies were incubated for 30 min at +4°C, washed with PBS and recovered in PBS prior to analysis with a LSRFortessa™ flow cytometer (Becton Dickinson). Data analysis was done with the FlowJo software.

### 2.9 Animal experimentations

Experiments were performed under the authorization number GE‐187–19 on groups of 6 animals. The fat was harvested by lipoaspiration with a blunt cannula from abdominal surgical samples of patient undergoing abdominoplasty (ethical authorization number GE 10–253). Pure fat was obtained after 3 min centrifugation by discarding liquid and oil. 0.5 ml of fat was pre‐mixed with 0.1 ml of ASC spheroids or single suspension ASC or saline buffer using a three‐way valve. 0.6 ml of human fat was placed through 1.05 mm cannula under the scalp skin of BALB/cJ mice under general anaesthesia with an inhalation of isoflurane 5% for induction and 3% for maintenance of anaesthesia throughout the study graft volumes were followed by a high resolution micro‐CT scan imaging (Quantum GX) under general anaesthesia. Data were analysed by a post‐processing software suite for image data (VivoQuant). Data are presented as mean ± standard error of the mean. The two‐way ANOVA was used for comparisons between groups. A *p* value of <0.05 was considered to indicate statistical significance. In another experiments, in the same way as before, human fat was injected with or without ASC expressing luciferase. Graft volumes were monitored over time by micro‐CT scan imaging (Quantum GX) and optical luminescence imaging (IVIS‐Spectrum) under isoflurane and 15 min after intraperitoneal injection of D‐Luciferin (VivoGlo™ Luciferin, In Vivo Grade, Promega) at 15 mg/ml. The dose was 10 µl/g of mice.

## 3 RESULTS

### 3.1 Manufacturing of individualized, homogeneous ASC spheroids by forced aggregation in agarose‐moulded microwells combined with air/liquid interface conditions

**FIGURE 1:**
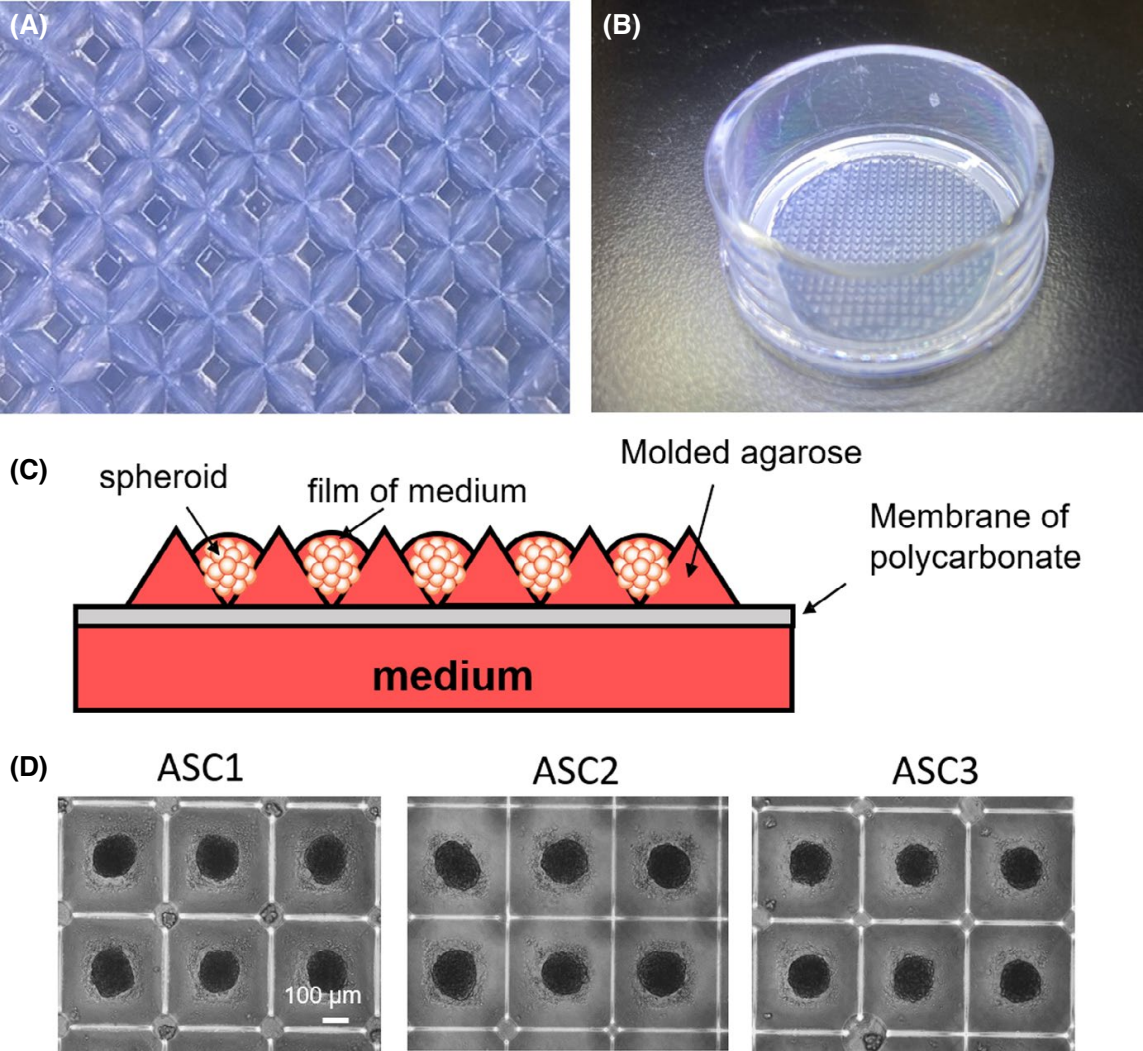
Forced aggregation of ASC in imprinted wells of agarose. (A) Microscopic view of imprinted agarose microwells. (B) Macroscopic view of a thin agarose mould on the hemi permeable membrane in its support. (C) Technical description of the method: forced aggregation in agarose combined with air/liquid interface. Spheroids are forced to aggregate, by centrifugation, in non‐adherent microwells. The agarose is on a polytetrafluoroethylene membrane that, by capillarity, attract the culture medium and create a thin film of medium on spheroids, the establishing air/liquid interface conditions. (D) Manufacturing of ASC spheroids of 3 different ASC cell line from 3 different patients by using the method described, after 48 h of aggregation

To manufacture ASC spheroids suitable for cell therapy in a large scale, forced aggregation is the most attractive method. However, ASC are highly adhesive cells and spheroids generated with this method attach to cell microplates, strongly limiting their homogeneity. To overpass these limitations, we generated non‐adherent microwells made of agarose (Figure 1A). Moulding of agarose was allowed by the intermediate manufacturing of pad of polydimethylsiloxane (PDMS), then used to create a footprint in heated agarose during jellification. Because immersion of microwells in culture medium favour hypoxia and the progressive occurrence of necrotic cores, we developed conditions to increase the exchanges between spheroids and the gas of air. Practically, the moulded agarose was very thin and formed at the surface of a polytetrafluoroethylene semipermeable membrane (Figure 1B), floating on culture medium to establish air/liquid interface conditions. In these air/liquid interface conditions, a thin film of medium then covers the spheroids. The hydrophilic and permeable nature of agarose allows nutrients transfer from the medium below the insert. The thin film of medium, compared to immersion of spheroids, provides more gas exchanges with air that could prevent the occurrence of necrotic cores and considerably improves the stability of each spheroid in their microwell (Figure 1C). Different ASC lines were assessed for the manufacturing of spheroids containing 1000 cells each (Figure 1D). The size of spheroids depends on the number of cells compacted in microwells. Other pads were also designed and 3D‐printed to create larger or smaller microwells, depending of the size of the suited spheroids and the number of needed cells. This variety of pads, and then microwells, allowed us to test several numbers of ASC per spheroid. Pads with large pyramids (Figure S1A) produced large microwells in agarose (Figure S1B,C). These larger microwells allowed the compaction of a high number of ASC, having tested 5000 cells and 10,000 cells, respectively (Figure S1D). We also manufactured spheroids using pads with small pyramids (Figure S1E), producing small microwells in agarose (Figure S1F,G). Smaller spheroids ranging from 250 cells to 1000 cells were manufactured in these smaller microwells (Figure S1H). In this study, focusing on the efficacy of ASC spheroids on fat transplantation, we made the choice to characterize spheroids containing 1000 ASC. Indeed, this number of cells created an optimal size suitable for injection through surgical needles and mixing with fat tissue.

Adipose‐derived stem cells spheroids after 48 h did not attach to the microwells and harboured a round shape (Figure 1D). The size of 30 spheroids per line was measured using ImageJ software, and the data were analysed (Table 1). For the all tested lines, the standard deviation was very low, under 5% of the diameter. Shape descriptors were analysed on 4 different ASC lines by the Fiji ImageJ software where six parameters were measured (*n* ≥ 28). All the parameters, including circularity, perimeter, aspect ratio, Feret's diameter, solidity and area, were highly homogeneous within the same line and among the different lines (Figure S2) with homogeneous values and low standard deviations. As ASC spheroids containing 1000 cells were stable and homogeneous after two days, and the importance to reduce time of manufacturing in clinical‐grade conditions, we characterized and transplanted ASC spheroids of 2 days.

**TABLE 1:** Spheroid diameter of 4 independant ASC lines (n=30) measured using ImageJ software (mean ± SD)

ASC line	9	10	11	12
Diameter in µm	166 ± 5.3	168 ± 4.3	159 ± 5.2	172 ± 4.5

### 3.2 Characterization of ASC spheroids and comparison with ASC in single cells.

**FIGURE 2:**
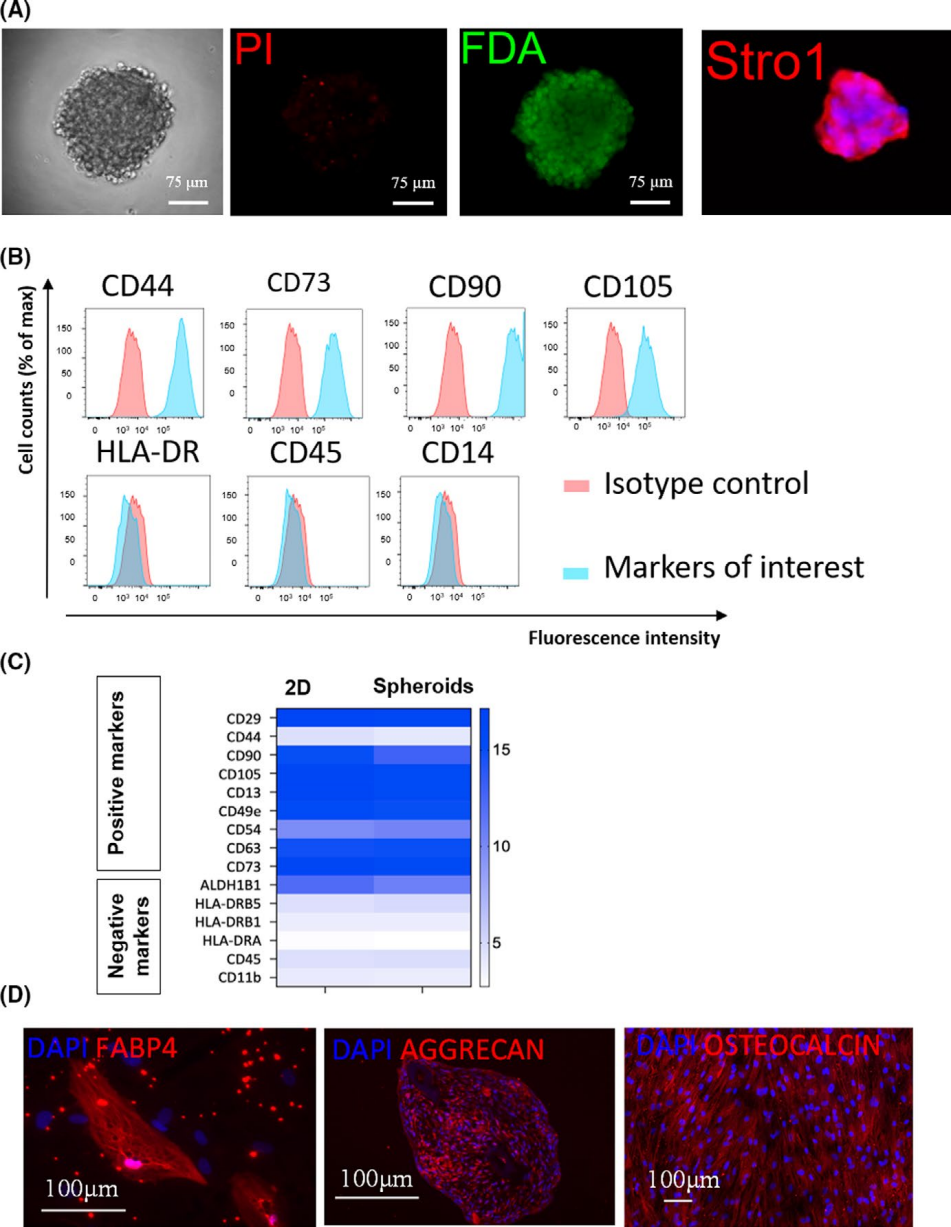
Characterization of the identity of spheroids made from ASC. (A) Staining of spheroids made from ASC with the method described in Figure 1D with FDA/PI or Stro‐1 antibody. (B) Flow cytometric analysis of ASC markers in single‐cell ASC derived from spheroids plated on plastic in tissue culture plates. (C) Comparison between spheroids and ASC in 2D for the mRNA expression of markers used for ASC identification. (D) Multilineage differentiation of single‐cell ASC derived from spheroids plated on plastic in tissue culture plates. Cells derived from spheroids were exposed to different media inducing differentiation towards adipocytes (FABP4), chondrocytes (Aggrecan) or osteocytes (Osteocalcine)

**FIGURE 3:**
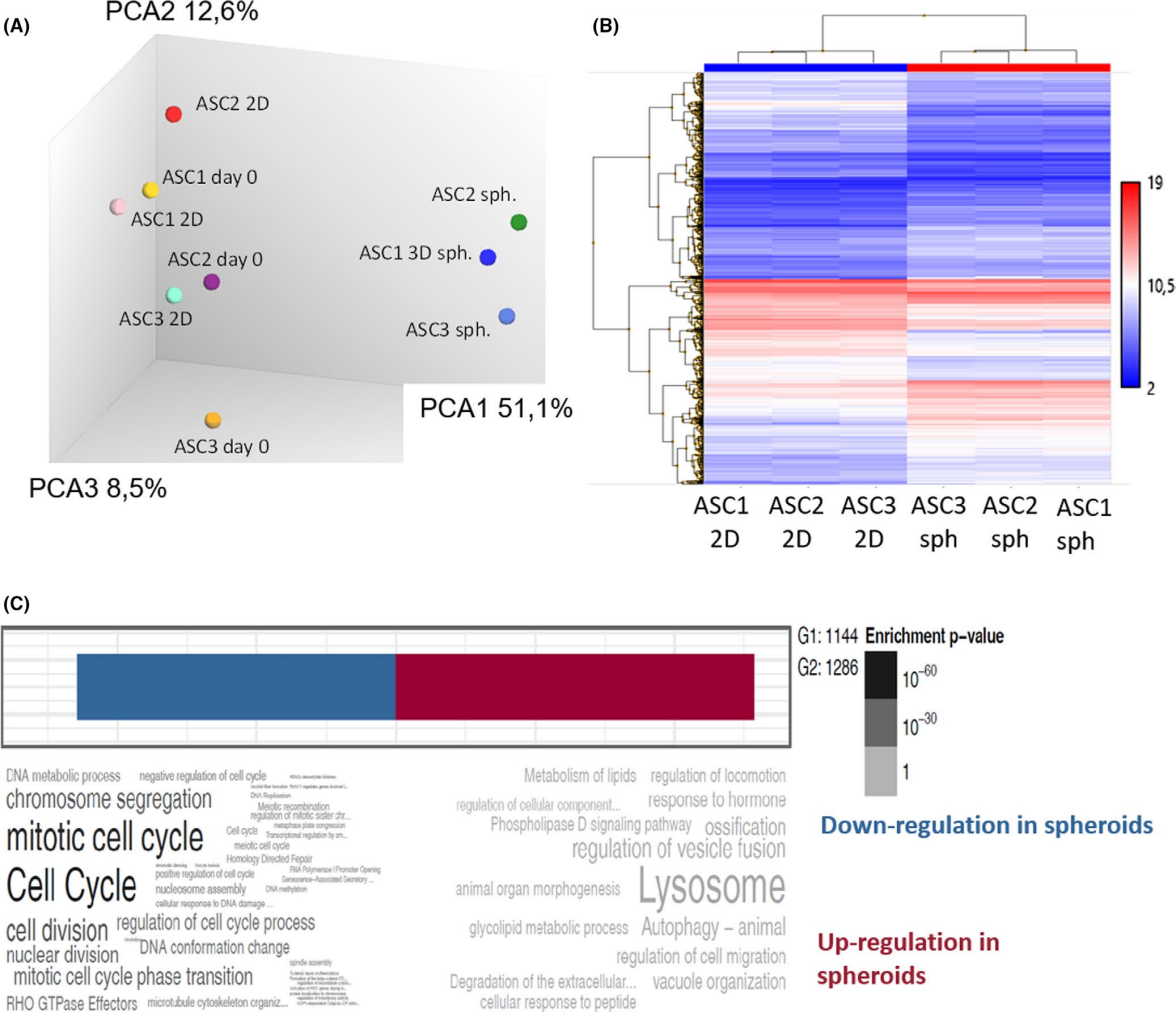
Gene regulation in spheroids compared to ASC in 2D. (A) The complete gene expression profile of ASC was analysed at day 0, or 48 h later in monolayer (2D) or spheroids, for three independent ASC lines (ASC1, ASC2 and ASC3) and compared through a principal component analysis. (B) Hierarchical clustering of complete gene expression profiles of 2 days ASC in 2D or 2 days spheroids derived from the same ASC. Three independent ASC lines are compared. (C) Word cloud analysis of the significant regulations in gene expression comparing 2 days ASC in 2D or 2 days spheroids derived from the same ASC. Three independent ASC lines are compared

To study the viability of spheroids made in these air‐liquid interface conditions, fluorescein diacetate and propidium iodide (FDA/PI) labelling was performed in 2 days spheroids. For this purpose, 30 randomly selected spheroids were co‐labelled with FDA/PI and showed viable cells without necrotic cores (Figure 2A). Spheroids made from ASC kept the expression of Stro‐1 after one week, a recognized tissue marker of MSC^35^ (Figure 2A). To investigate whether ASC in spheroids kept their phenotype and multipotency, spheroids were plated in conventional cell culture plates to derive rapidly single‐cell ASC directly analysable by flow cytometry. Cell‐surface analysis showed the expression of ASC markers (CD44, CD73, CD90 and CD105) and the absence of markers that must be negative in ASC (HLA‐DR, CD45 and CD14) in single‐cell ASC derived from one week spheroids (Figure 2B). On the other hand, a full gene expression profile was also performed in spheroids and compared with ASC in 2D conventional culture conditions. mRNA corresponding to ASC markers was found in both spheroids and 2D conditions, with the exception of CD44 (Figure 2C). Complete data of microarrays analysis are shown in Table S2. As CD44 expression was seen in flow cytometry (Figure 2B), we concluded to a weakness of CD44 detection in the microarray. Thus, cells in spheroids harboured a cell surface phenotype and gene expression profile compatible with an ASC identity. Multipotency of cells in spheroids made from ASC was also tested. The monolayer of cells derived from plated spheroids was differentiated, under appropriate media, towards adipocytes, chondrocytes and osteocytes, as seen by the expression of the adipocyte marker (FABP4), chondrocyte marker (aggrecan) and osteocyte marker (osteocalcin), respectively (Figure 2D). Thus, cell derived from spheroids made from ASC kept the multipotent properties of ASC. Next, we performed a microarrays analysis by using biological triplicates. Total RNA extracted from 3 independent ASC lines derived from three different patients were analysed on Affymetrix chips to compare the gene expression profile of ASC at day 0, or 48 h later in monolayer (2D) or spheroids. A principal component analysis (PCA) showed that the gene expression profile of ASC in spheroids differed from ASC in 2D or before their compaction (= day 0) (Figure 3A). A heatmap representing a global view of the level of expression of each transcriptome between the 2D and spheroids was also generated. The map generation software automatically ranked the conditions studied in order to group together the most concordant conditions. The heatmap (Figure 3B) classified automatically the 2D and spheroids into two well‐differentiated groups, confirming a gene regulation in spheroids compared to 2D. A functional analysis of the gene families most regulated by ASCs in spheroids was performed using an algorithm combining the statistical software R and the Gosummaries package. This generates a word cloud (Figure 3C) representing the functional families, with the most represented having a larger font size. All regulated genes with a fold change (between the 2D and 3D condition) >2 or <−2 and with statistical significance (FDR < 0.05) were considered (Table S1). Downregulated genes are below the blue part and up‐regulated genes are below the red part of the figure. Statistical significance is represented by a difference in contrast from light to dark grey in proportion to significance.

Genes related to cell cycle and cell proliferation were more frequently downregulated in spheroids whereas genes linked to autophagy (including lysosomal response) were upregulated. These observations suggested that ASC in spheroids reduced their proliferation compared to ASC in monolayers where proliferation and cell passaging are constant. An autophagic response to stress without toxicity (confirmed by viability assays described above) is also suggested.

A screening by arbitrarily selecting 215 genes involved in tissue regeneration, representing the fold change between 2D and spheroids, was performed. Ninety‐seven statistically significant regulations (based on the fold change >2 or <2 and *p* value < 0.05) were noticed (Figure S3). Among them, numerous genes responsible for cell proliferation were downregulated. MMP9, HGF, TGFβ3 and the angiogenic ANGPTL‐2 were notably upregulated whereas the pro‐inflammatory IL‐6 and CCL‐2 were downregulated. Of note, 3 genes coding for proteins of the extracellular matrix shows some spectacular upregulations: Dermatopontin (DPT), laminin alpha‐1 (LAMA4) and collagen 14 (COL14A1). Together, these data suggest the maintenance of a regenerative transcriptome in ASC spheroids, with some noticed regulations and a decreased proliferation. Total proteins were extracted from the supernatant of two different ASC lines (48 h) cultured for 12 h in protein‐free medium and analysed by mass spectrometry. The first difference noticed between spheroids and 2D conditions was the total amount of proteins found in the cell supernatant. Indeed, 15.51 ± 1.4 µg of protein per cell was found in the 2D samples. In contrast, in spheroids samples, only 6.92 ± 0.5 µg per cell were found, suggesting a barrier effect when cells are compacted or a mobilization in the extracellular matrix of spheroids.

**FIGURE 4:**
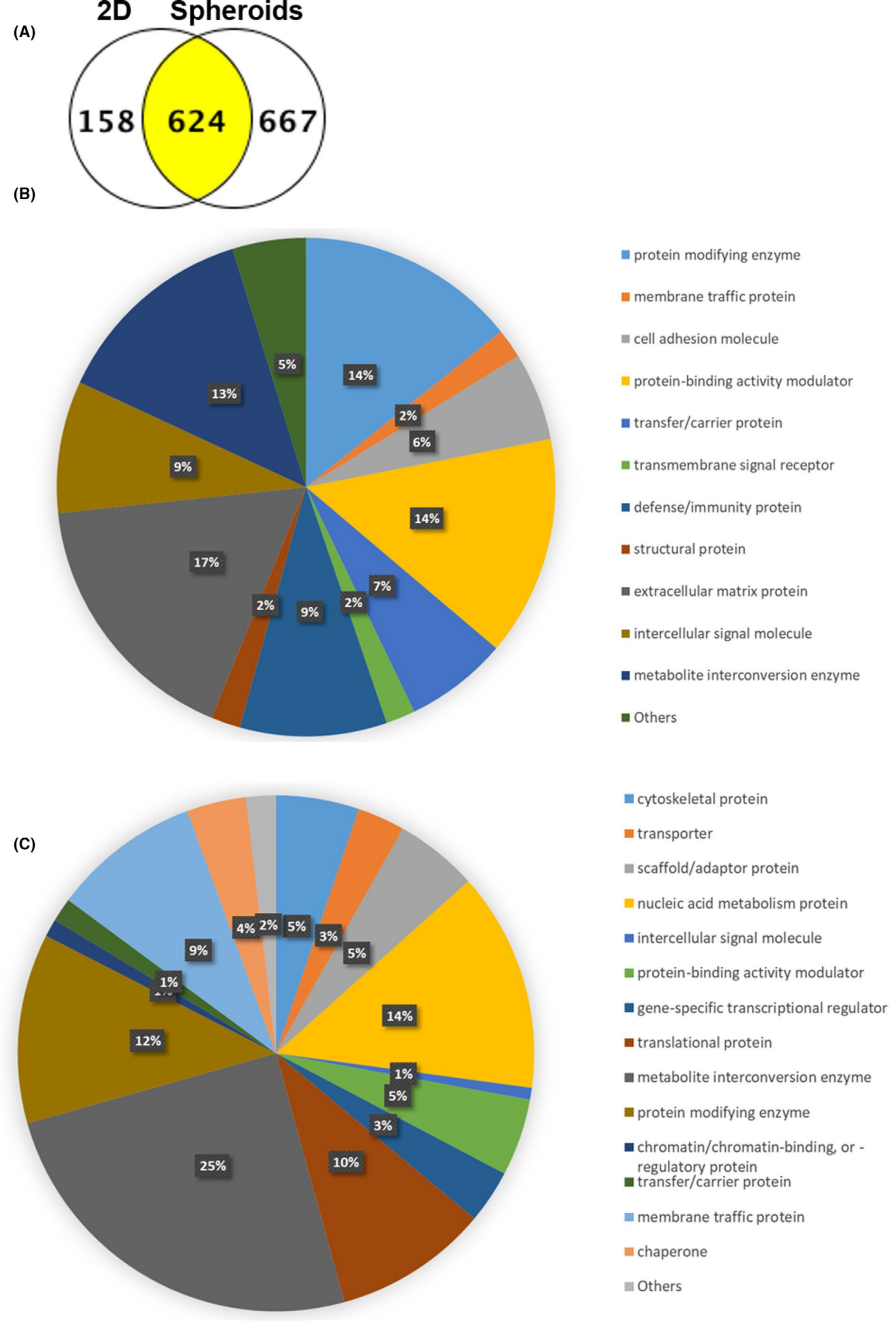
Analysis of the secreted proteome from spheroids versus ASC in 2D. (A) ASC in 2D or spheroids were cultured in serum‐free medium (=conditioned media) prior mass spectrometry analysis of its protein composition. The number of proteins identified in each sample is presented. (B) Functional protein class analysis was done by PANTHER (Protein ANalysis THrough Evolutionary Relationships) for proteins secreted only in 2D but not in spheroids. (C) Functional protein class analysis was done by PANTHER (Protein ANalysis THrough Evolutionary Relationships) for proteins secreted only in spheroids but not in 2D

In contrast, mass spectrometry identified a larger protein diversity in the spheroid samples compared to the 2D: 1291 proteins were identified in the spheroids samples whereas 782 proteins in the 2D samples (Figure 4A). Among them, 667 proteins were found only in the spheroids samples and 158 proteins were identified only in the 2D samples. Mass spectrometry data are shown in Table S3. Together, these observations show that the secreted proteome of ASC grown in spheroids was enriched in their diversity. Of note, some proteins were found from ASC in 2D but not secreted by the spheroids. To understand which proteins were not secreted after 3D formulation, a functional protein class analysis was done by the PANTHER (Protein ANalysis THrough Evolutionary Relationships) classification system. Among the proteins classes not found in the spheroid secretome, those from the extracellular matrix were mostly represented, probably highly mobilized in the 3D structure between compacted cells (Figure 4B). Other classes were also represented in this regulation, including ‘protein modifying enzymes’ and ‘protein binding activity modulator’. The reduced extracellular matrix protein secretion was confirmed by a string DB analysis, showing that among all the functional networks identified, one contained mainly collagens and extracellular matrix proteins (Figure S4). Another large functional network was also present, including heterogeneous cellular functions. On the opposite, the 667 proteins found only in the spheroids, but not in 2D, were classified in 16 functional groups (Figure 4C). Were mostly represented heterogeneous classes, including ‘metabolite interconversion enzyme, nucleic acid metabolism protein and protein modifying enzyme’ (Figure 4C). More generally, the enrichment of the secreted proteome in number and protein classes from ASC spheroids was probably due to the cell‐cell and cell‐matrix interactions and a more physiological environment closer to the in vivo situation.

### 3.3 Spheroids increase the engraftment of fat tissue in an animal model of lipofilling

**FIGURE 5:**
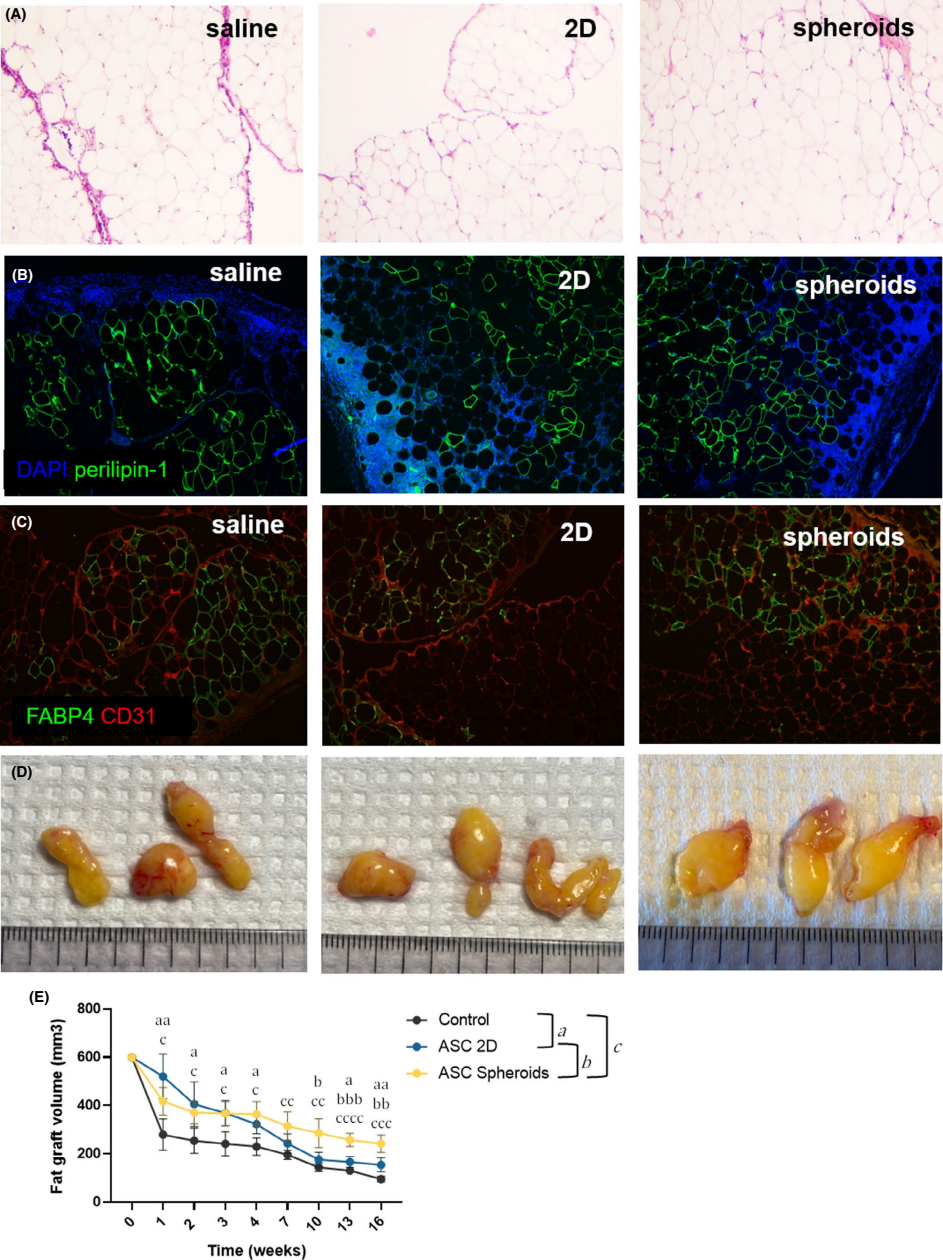
Regenerative properties of spheroids in an in vivo model of fat transplantation. (A) Hemalun/eosin coloration of fat transplants at week 7. (B,C) immunostaining with anti‐perilipin‐1, anti‐FABP4 and anti‐CD31 antibodies of fat transplants at week 7. (D) macroscopic view of fat transplants at week 7. (E) The size of fat transplants (*n* ≥ 3) was monitored after transplantation for 16 weeks by high resolution micro‐CT scan imaging. Two‐way ANOVA test statistics are indicated for each time point. Letters indicate significant differences between groups with a: comparison between Control and ASC 2D, b: comparison between ASC 2D and ASC spheroids and c: comparison between Control and ASC spheroids. Corresponding *p* values: a,b,c, *p* < 0.05; aa,bb,cc, *p* < 0.01; aaa,bbb,ccc, *p* < 0.001 and cccc, *p* < 0.0001

The regenerative properties of ASC spheroids were compared with ASC single‐cell suspensions in a mouse model of fat transplantation. The most recognized animal model for lipofilling is human fat subcutaneous transplantation in immunocompetent Balb/c mice.^36^ Immunosuppressed animals should not be used because the immune system and inflammation are a part of the regenerative process and fat immunogenicity is very low, with a delayed time for complete xenorejection. Human fat transplants were grafted subcutaneously in the scalp of Balb/c mice, with the addition of ASC in single‐cell suspension (2D) versus ASC spheroids versus control (vehicle alone). ASC stably transduced with lentivectors expressing firefly luciferase under the control of the ubiquitous short elongation factor promoter, allowing the monitoring of their viability after transplantation after intraperitoneal injection of D‐luciferin and live imaging in vivo showed that ASC spheroids were homogenously mixed in the fat transplant (Figure S5A). After 7 days, the luminescent signal strongly decreased in both conditions and was absent at day 11. Quantification of the luminescent signal in the grafts did not show differences between 2D and spheroids and a half‐life of 5 days in both conditions (Figure S5B), confirming a similar in vivo stability of spheroids and ASC in single‐cell suspension. The quality of fat transplants, assessed at week 7 by hemalum/eosin coloration was assessed in transplanted fat tissues showing no signs of necrosis (Figure 5A) and moderated leukocytes infiltration. The adipocyte markers perilipin‐1 and FABP4 were homogeneously expressed in all conditions, confirming the quality of transplants at week 7 (Figure 5B,C). At week 16, xenorejection was more intense, with a very high infiltration of the fat tissue by reactive leukocytes and small grafts with necrotic areas (not shown). Macroscopically, transplants showed a compact aspect and an expected yellow colour with a certain degree of vascularization (Figure 5D), without any microscopic change in vessel formation in the condition with ASC spheroids, regarding the expression of CD31 (Figure 5C). At week 7, the macroscopic size of transplants was higher in the presence of ASC than control conditions with vehicle alone (Figure 5D). Groups of animals for each condition were monitored in vivo by CT scan imaging during 16 weeks and the volume of grafts determined by image analysis in 3D (Figure 5E). The long‐term stability of transplants was significantly increased with ASC spheroids compared to ASC in single‐cell suspension starting at week 10 post‐transplantation and maintained until week 16 post‐transplantation, showing an increase, on average, of 25% of the size of transplant's size between week 10 and week 16. Together, these observations confirm that ASC spheroids kept their stability in vivo after transplantation with human fat tissue. Importantly, the increased volume of transplants enriched with spheroids is associated with a histological aspect of healthy fat tissue and not over inflammatory tissue with oedema and increased leukocyte infiltration, indicating that transplants got less resorbed with the expected quality.

## 4 DISCUSSION

The new method described in this study aimed at overpassing all of the current limitations to produce ASC spheroids, combining advantages of dynamic and static conditions, without any external scaffold. Its simplicity and very high level of standardization provide a realistic possibility for the transition to clinical application. Forced aggregation in designed microwells is the best option to rapidly form highly homogeneous ASC spheroids. The moulding of microwells in agarose prevents adhesion of spheroids to their support. At the same time, agarose is hydrophilic and allows permanent diffusion of nutrients towards the spheroids. Importantly and in contrast to dynamic immersion, air/liquid interface conditions (i) stabilize each spheroid in their microwell allowing medium change on the other side of the membrane and (ii) provide optimal gas exchanges with air to prevent the development of necrotic cores in static conditions.

An important question was the identity of cells within the spheroids. According to the International Federation for Adipose Therapeutics and Science (IFATS) definition criteria, cell in spheroids corresponded to an ASC identity: cell surface phenotype and genotype, adherence to plastic and multipotency.^5^ Interestingly, the full gene expression profile ASC spheroids differed from ASC in single‐cell suspension but kept its regenerative properties. Additional comparisons with the gene expression profile of adult human fibroblasts did not confirm any similarities (data not shown), thus excluding fibroblastic differentiation of ASC in spheroids. Cell proliferation was reduced in ASC spheroids compared to monolayers. Direct toxicity due to the absence of nutrients/oxygen in spheroids seems unlikely because necrotic cores were not observed and FDA/DAPI analyses confirmed the viability of all cells within the aggregates. As it has been demonstrated that human collagen inhibits MSC proliferation,^37^ and having confirmed the increased collagen production by ASC in spheroids, we hypothesized that the decreased proliferation was linked to cell‐to‐cell or cell‐to‐matrix interactions. Moreover, and more generally, cells in more hypoxic zones of spheroids have a decreased proliferation.^38^ Stemness markers (Nanog, Sox‐2, Oct‐4) were however not altered in ASC spheroids (data not shown), making the selection of stem cell niches in these conditions unlikely. Transcriptome and secretome data suggest an increase of cell autophagy in spheroids compared to monolayers. Autophagy generally helps the cells to adapt to stress conditions and maintain their homeostasis,^39^ thereby enabling the tissues to maintain a controlled growth and development. Hence, cores in ASC spheroids could induce adaptative autophagic response to an hypoxic or metabolic stress to maintain the cell integrity and viability. Autophagy is a major inducer of vessels formation^40^ and was shown to protect MSC from apoptosis under hypoxic conditions.^41^ Moreover, although not confirmed in our study, autophagic MSC were described in some studies to accelerate regeneration.^40^

The regenerative secretome of ASC spheroids is thought to play a critical role in cell therapy applications. The first observation is that ASC spheroids, compared to the same number of cells in monolayer, have quantitatively a reduced total secreted proteome outside the spheroids. However, the protein diversity is higher. The question of the secreted proteome from spheroids remains ambiguous: with monolayers, proteins are secreted in the cell culture medium. With spheroids that produce their own extracellular matrix, a part of secreted proteins will enter into the matrix. Thus, it could explain the reported lower concentration of extracellular matrix proteins in the culture medium compared to 2D. The reduced secretion of proteins could also be explained by physical barriers to protein diffusion. Proteins produced by cells in the core of spheroids must diffuse and cross several layers with active processes and receptors, in contrast to 2D where proteins are directly and immediately released in the medium. Thus, the multicellular organization of spheroids could filter and then reduce the release in the medium. Also, cell regulations in channels for cell‐to‐cell proteins transport could be altered but were not seen in our transcriptome data (not shown). In addition, mounting evidence reveals that autophagy reduces protein secretion.^42^ Interestingly, protein diversity in the conditioned medium was higher with spheroids, suggesting cell regulations. No clear functional families were identified within the additional secreted proteome induced by spheroidal formation. From a therapeutic point of view, only in vivo data on regeneration could indicate if this protein diversity in the secreted proteome could have a positive impact for therapy.

The in vivo experiments in this study confirmed a therapeutic advantage of spheroids made with this method compared to ASC formulated in single‐cell suspension. Autologous fat grafting is increasing in popularity for breast reconstruction after cancer treatment; in the treatment of burn scars, congenital malformations and post‐traumatic malformations. The major obstacle is an unpredictable and often low graft survival, with resorption rates ranging from 25% to 80%.^11^ The beneficial effect of spheroids compared to single cells on graft survival is thought to be due to their secretome. Their survival in transplants was not significantly different from ASC in 2D, although twofold superior at day 7 post‐transplantation but strongly reduced compared to day 0 (Figure S5B). No signs of increased vascularization were seen with spheroids compared to 2D (hemalun/ eosin coloration and CD31 staining, Figure 5) and scratch test in vitro did not show any increased fibroblastic proliferation with medium conditioned with ASC spheroids (not shown). The expected xenogenic reaction, visualized by a progressive invasion of human fat with mouse leucocytes, did not differ between ASC in 2D or spheroids (not shown). Together, these observations suggest that the beneficial effects of spheroids are not due to angiogenic factors, immunomodulatory or trophic effects. Accordingly, Kolle et al assessed clinically human ASC in single‐cell suspension during autologous fat transplantation and found no difference in vessel density between the ASC‐enriched and non‐enriched grafts^11^ and suggested that the co‐implanted ASC might have differentiated into fat cells. The lack of luciferase activity in our grafts in all conditions does not confirm this hypothesis. The main differences observed between ASC in 2D and spheroids are the protein diversity of their secretome and the upregulation of extracellular matrix gene expression, especially some collagens. Besides their structural functions, collagens play a pivotal role as a signalling molecule in the regulation of all phases of healing. Notably, peptides released by proteolytic degradation of collagens exhibit chemotactic properties and facilitate cellular differentiation, migration and recruitment of macrophages and fibroblasts.^43^

We hypothesize that the therapeutic effect of ASC spheroids is mediated by their secretome and possibly production of extracellular matrix. Regarding the clear advantage of MSC spheroids for regeneration in many preclinical reports,^13^, ^15^, ^16^ further studies are required to better understand the mechanisms of action explaining their advantage.

## 5 CONCLUSION

In conclusion, ASC spheroids have a clinical potential to increase the engraftment of fat tissue more efficiently than ASC formulated in single‐cell suspension.

## CONFLICT OF INTEREST

The authors declare that they do not have any conflict of interest.

## AUTHOR CONTRIBUTIONS

**Sanae El‐Harane:** Conceptualization (lead); Data curation (lead); Formal analysis (lead); Methodology (lead); Visualization (lead); Writing – original draft (equal); Writing – review & editing (equal). **Stephane Durual:** Conceptualization (equal); Methodology (equal). **Thomas Braschler:** Conceptualization (equal); Methodology (equal). **Dominik André‐Lévigne:** Methodology (equal); Resources (equal). **Nicolo Brembilla:** Formal analysis (equal); Methodology (equal). **Karl‐Heinz Krause:** Data curation (equal); Formal analysis (equal); Methodology (equal); Resources (equal). **Ali Modarressi:** Conceptualization (lead); Data curation (lead); Formal analysis (lead); Funding acquisition (lead); Methodology (lead); Resources (lead); Supervision (lead). **Olivier Preynat‐Seauve:** Conceptualization (lead); Data curation (lead); Formal analysis (lead); Funding acquisition (lead); Methodology (lead); Supervision (lead); Writing – original draft (lead).

## Supporting information

Fig S1

Fig S2

Fig S3

Fig S4

Fig S5

Table S1

Table S2

Table S3
