# Supplementary figures and images for: Adipose‐derived stem cell spheroids are superior to single‐cell suspensions to improve fat autograft long‐term survival
Source: J Cell Mol Med. 2022 Feb 11;26(5):1421–33. doi: 10.1111/jcmm.17082 (PMC8899177; doi:10.1111/jcmm.17082)

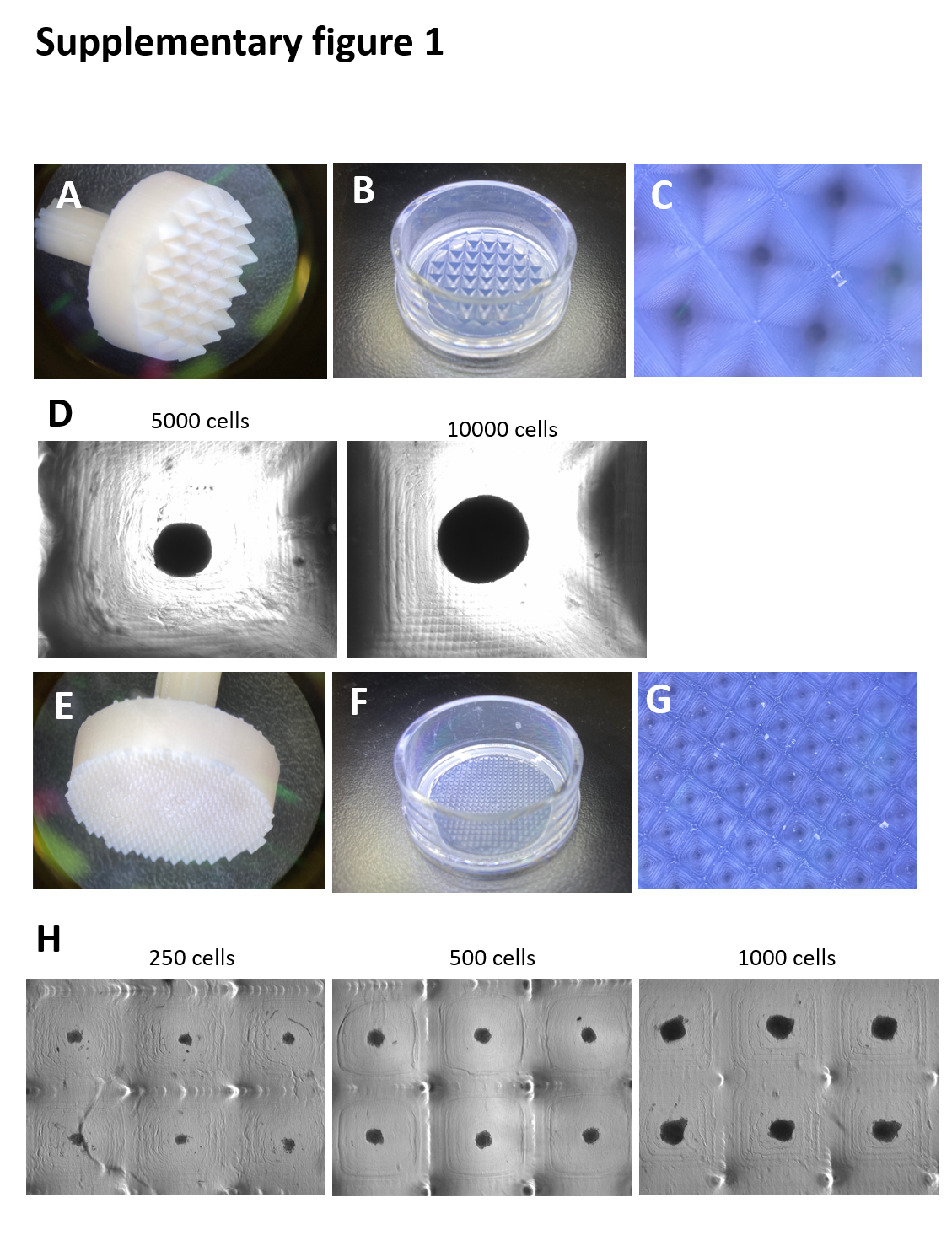

Supplement: Supplementary file 1 — Fig S1 [file JCMM-26-1421-s004.png]

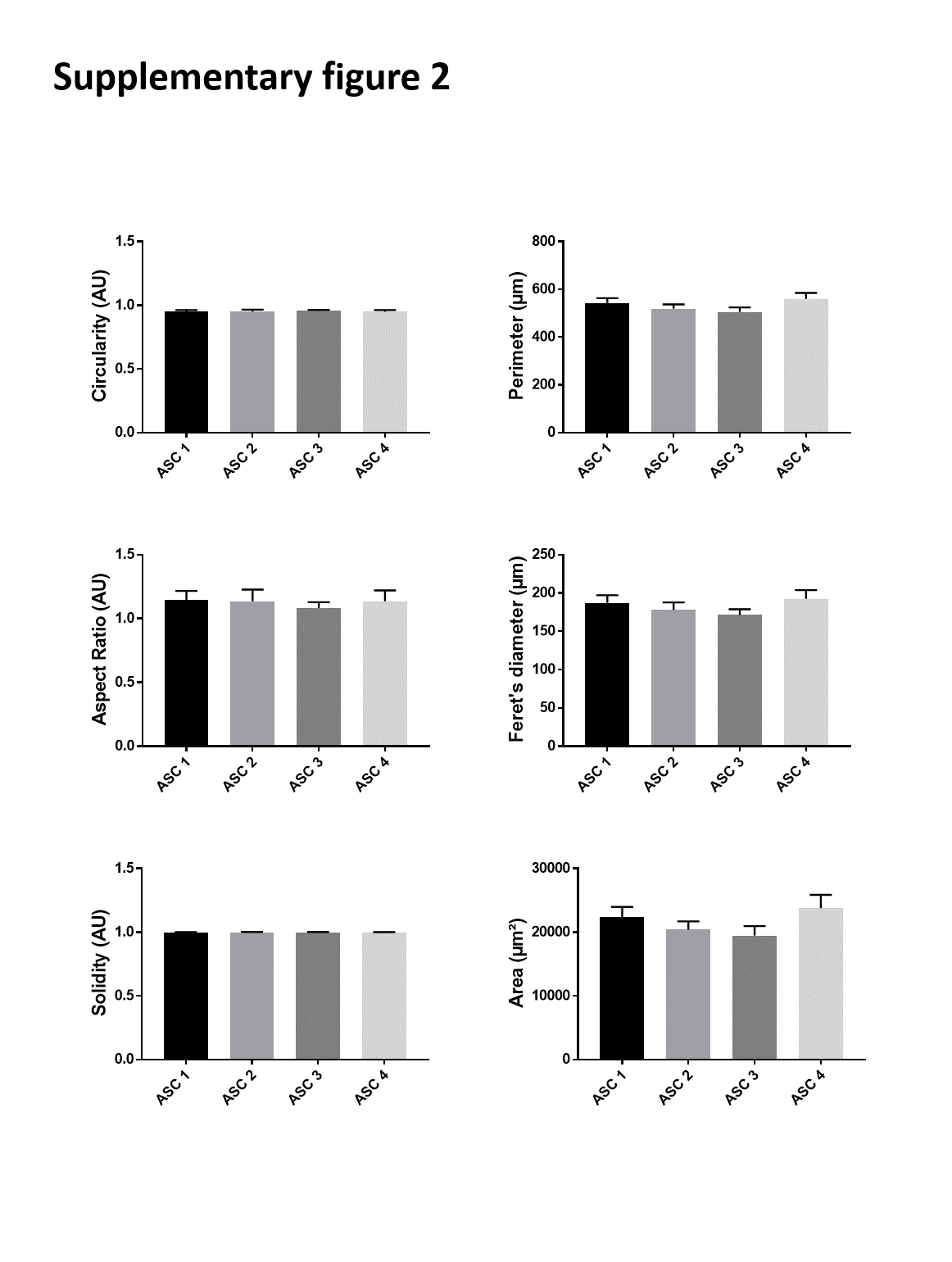

Supplement: Supplementary file 2 — Fig S2 [file JCMM-26-1421-s005.png]

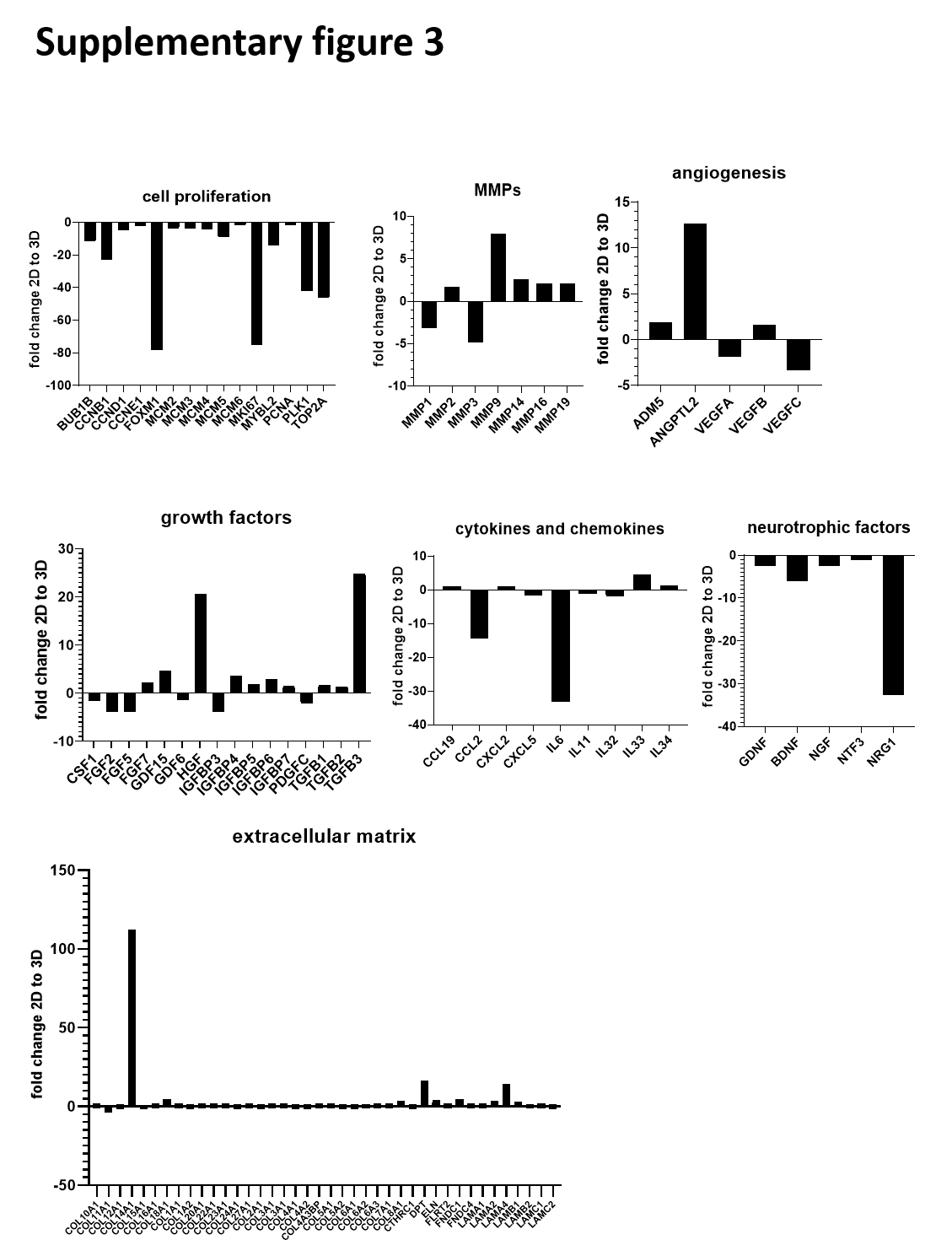

Supplement: Supplementary file 3 — Fig S3 [file JCMM-26-1421-s007.png]

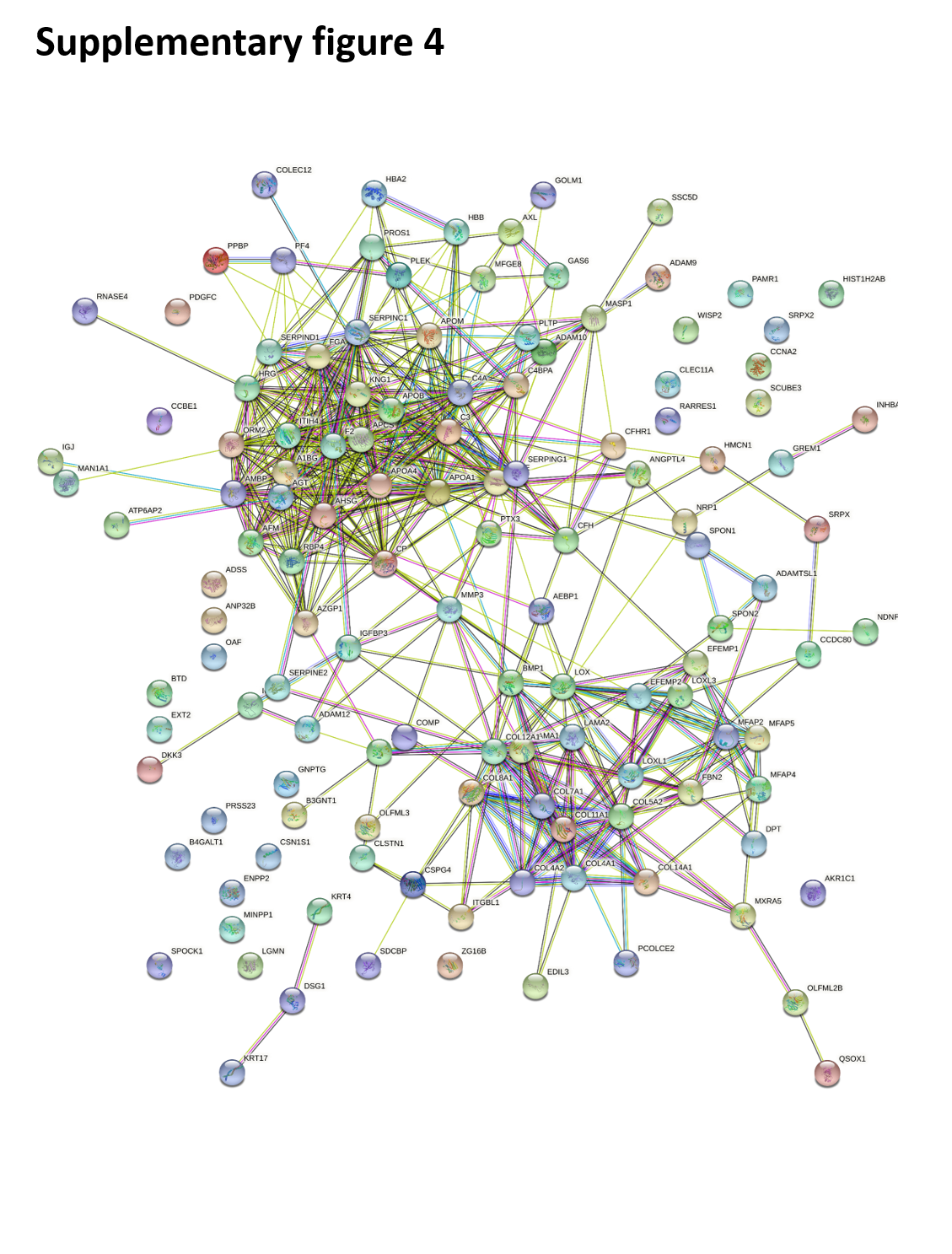

Supplement: Supplementary file 4 — Fig S4 [file JCMM-26-1421-s003.png]

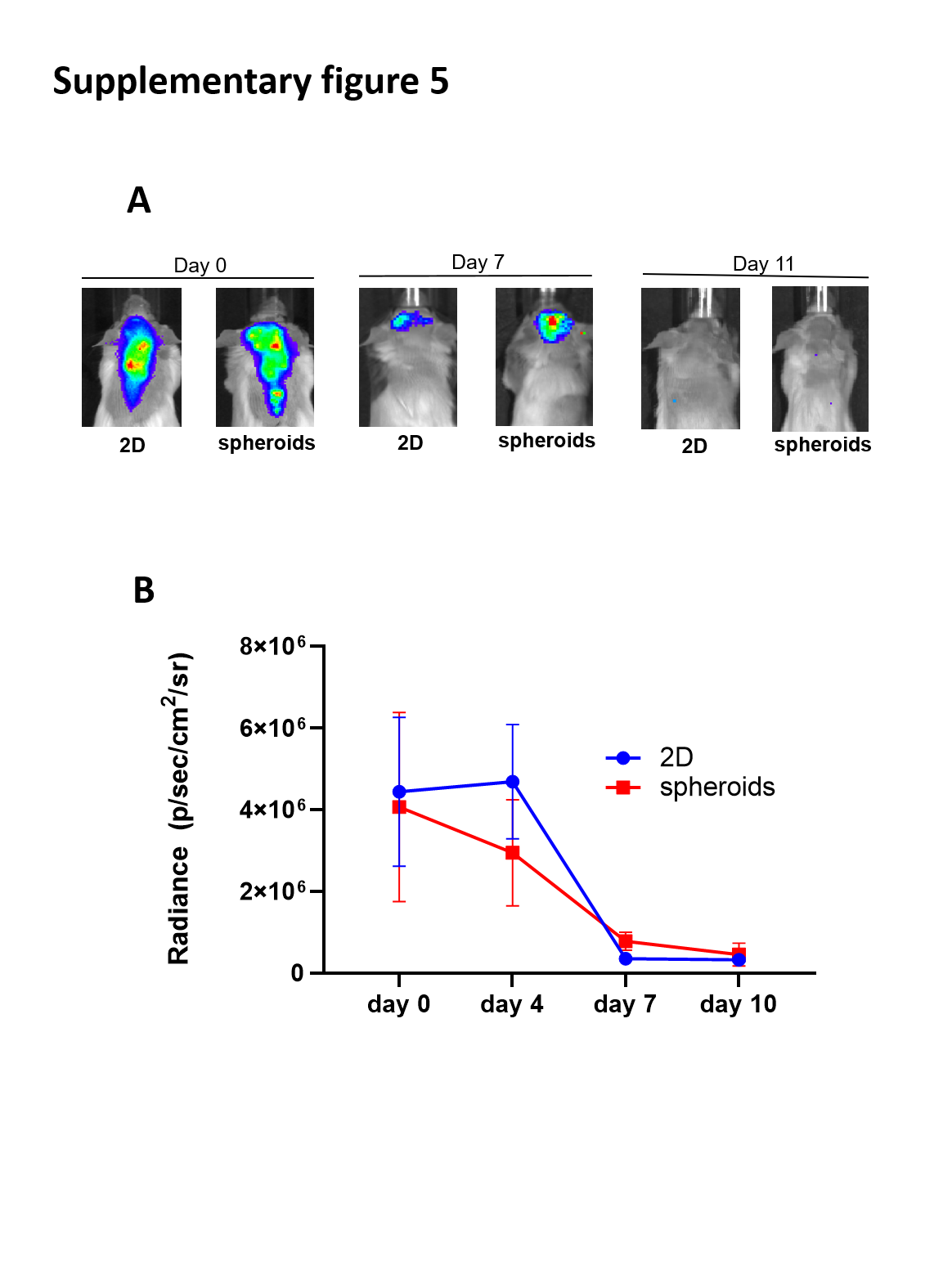

Supplement: Supplementary file 5 — Fig S5 [file JCMM-26-1421-s001.png]
